# Mapping short tandem repeats for liver gene expression traits helps prioritize potential causal variants for complex traits in pigs

**DOI:** 10.1186/s40104-021-00658-z

**Published:** 2022-01-17

**Authors:** Zhongzi Wu, Huanfa Gong, Zhimin Zhou, Tao Jiang, Ziqi Lin, Jing Li, Shijun Xiao, Bin Yang, Lusheng Huang

**Affiliations:** State Key Laboratory for Pig Genetic Improvement and Production Technology, Jiangxi Agricultural University, Nanchang, China

**Keywords:** Cis-eQTL, Co-localization, Gene expression, Liver, Pig heterogeneous population, Short tandem repeats

## Abstract

**Background:**

Short tandem repeats (STRs) were recently found to have significant impacts on gene expression and diseases in humans, but their roles on gene expression and complex traits in pigs remain unexplored. This study investigates the effects of STRs on gene expression in liver tissues based on the whole-genome sequences and RNA-Seq data of a discovery cohort of 260 F6 individuals and a validation population of 296 F7 individuals from a heterogeneous population generated from crosses among eight pig breeds.

**Results:**

We identified 5203 and 5868 significantly expression STRs (eSTRs, FDR < 1%) in the F6 and F7 populations, respectively, most of which could be reciprocally validated (π1 = 0.92). The eSTRs explained 27.5% of the cis-heritability of gene expression traits on average. We further identified 235 and 298 fine-mapped STRs through the Bayesian fine-mapping approach in the F6 and F7 pigs, respectively, which were significantly enriched in intron, ATAC peak, compartment A and H3K4me3 regions. We identified 20 fine-mapped STRs located in 100 kb windows upstream and downstream of published complex trait-associated SNPs, which colocalized with epigenetic markers such as H3K27ac and ATAC peaks. These included eSTR of the *CLPB*, *PGLS*, *PSMD6* and *DHDH* genes, which are linked with genome-wide association study (GWAS) SNPs for blood-related traits, leg conformation, growth-related traits, and meat quality traits, respectively.

**Conclusions:**

This study provides insights into the effects of STRs on gene expression traits. The identified eSTRs are valuable resources for prioritizing causal STRs for complex traits in pigs.

**Supplementary Information:**

The online version contains supplementary material available at 10.1186/s40104-021-00658-z.

## Background

Expression quantitative trait loci (eQTL) mapping studies identify DNA variants linked to gene expression traits, is a powerful approach for identifying target genes that mediate the effects of genetic variations on complex traits [1]. To date, most genome-wide association mapping studies on gene expression traits have focused on SNPs and InDels, while other forms of mutations such as Short tandem repeats (STRs) and structural variations, have been ignored, leading the QTLs driven by these variants to be overlooked.

STRs are highly polymorphic genetic markers with repeat unit lengths of 1–6 base pairs that have been widely used in population genetics, forensic medicine, and aetiology research [2]. Abnormal STR mutations may cause human disease. For example, the (CCG)n repeats in the promoter region of the *FMR1* gene were found to disrupt the chromatin topology domain (TAD) structure and DNA methylation, thereby altering the expression of *FMR1* and causing Fragile X Syndrome [3]. Associations of STRs with phenotypic traits have also been reported in non-human species, including associations between a (GT)n polymorphism in the 3’UTR of the *SLC11A1* gene and resistance to brucellosis in bovines [4]; a (GAA)n amplification in the intron of the *ITPR1* gene and progressive gait abnormalities in dogs [5] and (AAGAG)n-rich RNA in Drosophila and the sperm maturation process after meiosis [6]. Recently, STRs were found to have a significant impact on gene expression traits in both humans [7–9] and plants [10, 11], and the results of association analyses of STRs with gene expression traits provide valuable resources to prioritize causal variants for complex traits.

Pigs (*Sus scrofa*) are important agricultural animals and a good model organism for biomedical research. STR markers have been widely used as genetic markers to survey population structure [12], genetic diversity [13], pork breed traceability [14] and QTL mapping [15] in pigs. Recent studies have shown that (AC)n repeats in the porcine *IGF1* gene promoter affect the binding of HIF1α and the expression of *IGF1* [16] and that (AC)n repeats in the promoter of the *SIX1* gene are significantly associated with carcass weight and backfat thickness [17]. However, few studies have investigated genome-wide STR effects on gene expression and used the results to dissect the potential causal variants underlying complex traits.

**Fig. 1:**
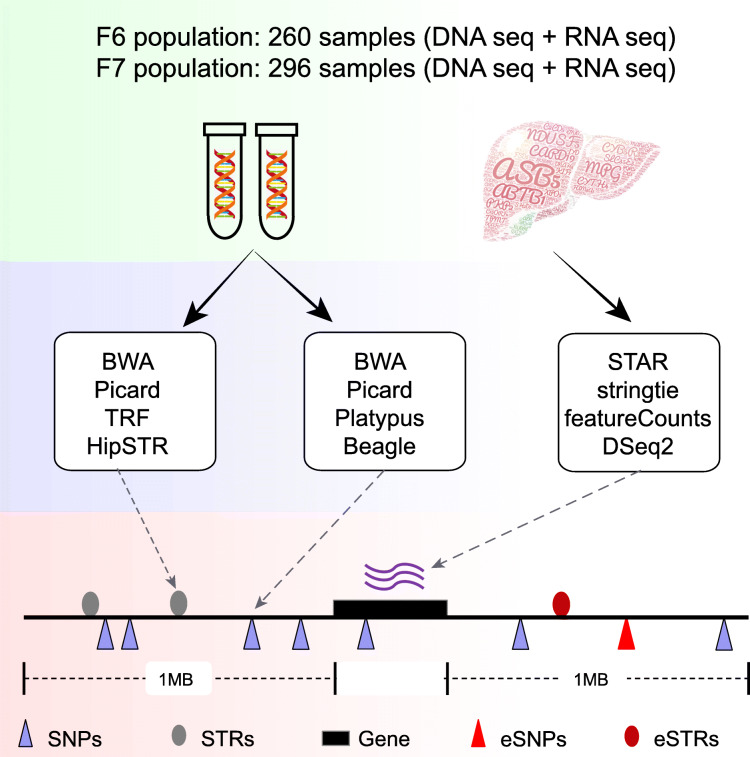
Schematic representation of the cis-region eSTR analysis. Red circles represent significant eSTRs and red triangles denote significant eSNPs. The association analysis was performed within 1 Mb windows on either side of each gene

Here, we assembled a dataset consisting of whole-genome sequencing (WGS) and liver transcriptome data from 260 F6 and 296 F7 individuals from a heterogeneous pig population (Fig. 1; Additional file 1: Fig. S1) to identify STRs with significant effects on the gene expression (eSTRs). We used a Bayesian fine-mapping approach to quantify the probability that each eSTR was the causal variant [18] and defined STRs with a posterior probability greater than 0.1 as candidate fine-mapped eSTRs. We characterized the overlap of genomic features and epigenomic markers with eSTRs and fine-mapped eSTRs. Moreover, we identified a number of fine-mapped eSTRs within 100 kb windows near published GWAS signals, among which 20 overlapped with epigenomic signals marking promoters, enhancers or other open chromatin regions. This work reports a comprehensive eSTR analysis of pig liver tissue, which is valuable for dissecting the causal STRs and their target genes underlying complex trait loci in pigs.

## Materials and methods

### Ethics statement

All experiments on pigs were performed under the guidance of Jiangxi Agricultural University, Nanchang, China.

### Samples and data

The animals investigated in this study included 260 F6 and 296 F7 individuals from a heterogeneous pig population generated by crossing eight founder breeds, including Bamaxiang (BX), Erhualian (EH), Laiwu (LA), Tibetan pig (TB), Duroc (DU), Landrace (LD), Large White (LW) and Pietrain pigs (PT). We designed a circular mating program to equally mix the ancestries of the eight founder breeds in individuals of the third and later generations [19]. Each founder breed was therefore expected to contribute 12.5% of the genetic ancestry in each F6/F7 individual. All F6/F7 individuals were raised on a pig farm in Nanchang, Jiangxi Province, China; boars were castrated at 90 ± 10 d. Subsequently, all individuals were fed ad libitum under Osborne’s FIRE^®^ (Feed Intake Recording Equipment) System (Osborne Industries, Inc., Osborne, Kansas, USA). The *longissimus* muscle and liver tissues were collected after slaughter at 240 ± 10 d. The collected samples were frozen in liquid nitrogen immediately after slaughter within 30 min and were stored in a refrigerator at − 80 °C.

DNA was extracted from the *longissimus* muscle using the standard phenolic chloroform method. The DNA concentration and quality of all samples were assessed using a NanoDrop-1000 instrument (Thermo Fisher Scientific., Waltham, Massachusetts, USA) and agarose gel electrophoresis (0.8%), respectively. DNA library construction and sequencing were performed independently for each sample at Novogene Bioinformatics Technology Co., Ltd. (Beijing, China). In brief, genomic DNA was broken into 300–400 bp DNA fragments and then amplified by Polymerase chain reaction (PCR) using Phusion^®^ High-Fidelity DNA polymerase Kit (New England Biolabs, Inc., Massachusetts, USA) following the manufacturer’s protocol. The libraries were sequenced on an Illumina HiSeq Xten platform (Illumina, Inc., San Diego, California, USA) by generating 2 × 150 bp paired-end reads. On average, the sequencing depth for each individual was approximately 8 ×. Low-quality DNA reads that contained more than 10% N bases or 50% low-quality bases (Q ≤ 5) were filtered out. Total RNA was extracted from liver tissue using TRIzol™ (Invitrogen, Carlsbad, California, USA). RNA purity, integrity and concentrations were measured using an eNanoPhotometer^®^ spectrophotometer (Implen, Inc., Calabasas, California, USA), a Bioanalyzer 2100 system (Agilent Technologies, Inc., Santa Clara, California, USA) and a Qubit3.0 Fluorometer, respectively. RNA sequencing libraries for each sample (F6 and F7) were constructed with the NEBNext^®^ UltraTMR NA Library Prep Kit for Illumina (New England Biolabs, Inc., Massachusetts, USA) according to the manufacturer’s specifications. In short, mRNA was purified from 2-μg total RNA from each sample using Oligo (dT) magnetic beads (Invitrogen, Carlsbad, California, USA) and was then fragmented with fragmentation buffer (Ambion, Inc., Austin, Texas, USA). Next, the random primers and reverse transcriptase (Invitrogen, Carlsbad, California, USA) were used for cDNA synthesis. The cDNA was then purified, end-repaired, adaptor-ligated, and amplified following the recommendations of the manufacturer. Finally, the RNA-seq libraries of F6 and F7 individuals were sequenced on the Illumina HiSeq Xten PE150 platform (Illumina, Inc., San Diego, California, USA) and the Novaseq-6000 PE150 platform (Illumina, Inc., San Diego, California, USA), respectively. The raw RNA reads with the percentage of low quality (Q ≤ 5) bases greater than 50% and N base contents greater than 10% were removed, generating an average of 12.74 Gb data for each individual.

### Genotyping and filtering

We used Tandem Repeat Finder (TRF v4.6 [20]) to scan candidate STR regions in the pig Sscrofa 11.1 reference genome (http://asia.ensembl.org/Sus_scrofa) using the parameters “TRF –80, 10, 6, 2”. We retained STRs with at least three repeating units and removed redundant and complex STRs. In addition, adjacent STRs separated by less than 10 bp were also filtered out. A total of 1.72 million STRs consisting of 962,775 single-nucleotides, 267,217 dinucleotides, 75,813 trinucleotides, 75,813 tetranucleotides, 90,161 pentanucleotides, and 92,437 hexanucleotides were identified. We used the FastQC program to evaluate the quality of the DNA-seq data, BWA-MEM to map the sequences to Susscrofa11.1, Picard software to mark duplicated PCR reads and Samtools [21] to sort and index the generated BAM files. We used Platypus [22] to identify SNPs and InDels. After filtering out low quality variants (MAF < 0.05 and call rate < 0.8), Beagle4 software was used to impute the missing SNPs and InDels [23]. A total of 19,465,449 SNPs and 5,107,941 InDels were detected in 556 individuals.

HipSTR software [24] was used to call the STR genotypes of all individuals jointly. We further filtered out STRs with an average sequencing depth of less than 5, an average quality value of less than 0.9, a length greater than 150 bp, an average heterozygosity in 556 F6/F7 samples of less than 0.1, and those for which the genotype was detected in less than 100 individuals. Finally, 330,205 STR loci that satisfied the above conditions were retained for subsequent analysis. Overall, an average of 175,972 STR loci were detected per individual, and an average of 297 samples were detected for each locus (Additional file 2: Fig. S2).

### Gene expression

We used FastQC software to evaluate the quality of the RNA-Seq data and the STAR program [25] to map the clean reads to the reference genome. By referring to the GFF annotation of the ensemble database (ftp://ftp.ensembl.org/pub/release-97/gff3/sus_scrofa), the liver tissue transcripts were assembled using StringTie [26] and quantified with featureCounts software [27]. Genes with overall raw expression counts of less than 30 in the 556 individuals were excluded. Finally, DESeq2 [28] was used to standardize gene expression values, for which the fragments per kilobase of exon model per million mapped reads (FPKM) method was adopted in this study. A total of 18,684 single-copy genes were retained for analysis.

### eSTR analysis

The eSTR analysis was performed in the F6 and F7 populations separately. We focused on STRs within 1 Mb from the corresponding genes. We corrected the gene expression data for sex, age, breeding batch, carcass weight, RNA integrity numbers (RINs) for RNA-seq, the top 10 PCs based on genome-wide SNPs, and 20 peer factors inferred from the expression data [29]. All genotype data and corrected expression data were normalized using a scale function with the default parameters in R, respectively. Finally, we used the ordinary least squares method (OLS) in the statsmodels package to analyse the associations between STR genotypes and gene expression levels. To compare the effects of STRs and SNPs on gene expression, we also performed eQTL analysis of the SNPs within ± 1 Mb from corresponding genes using the same pipelines.

### Significance thresholds

The multiple testing correction was performed using an approach that integrates Bonferroni and Bengamini hochberg method [8, 9]. For each gene, we first corrected the *P* value of each gene-STR test according to the Bonferroni method and retained the most significant STR for each gene. Then, all adjusted *P* values were corrected by the Benjamini-Hochberg method using the qvalue package, and the threshold of significance was set to an FDR = 1%.

### Variance component analysis of eSTRs

We fit two models (H0: Expression = SNP + errors vs. H1: Expression = SNP + eSTR + errors), and then determined whether the two regression equations were significantly different using the Python based on a significance threshold of an FDR < 5%. The SNP data were processed by using “plink --indep 50 5 2” to delete strongly linked sites. The phenotypic variance was considered to be the sum of genetic variance attributed to an eSTR, the surrounding SNPs and the residuals: V(p) = V(eSTR) + V(SNP_all_) + V(e). The heritability of an eSTR was calculated as h_STR_ = V(eSTR) / V(p), and the heritability of a SNP was computed as h_SNP_ = V(SNP_all_) / V(p). In the model employed for variance component analysis, the eSTR was considered a fixed effect, the aggregate effect of the other cis-SNPs was considered a random effect, and the cis-heritability was dissected using GCTA software [30]. Both the expression matrix and genotype matrix were standardized before variance component analysis.

### Fine-mapping of causal variants

In this study, CAVIAR (V2.2) software was used to further fine map eSTR signals. All significant eSNPs (*P* < 0.001) and eSTRs (FDR < 1%) were considered as the candidate variant sets, and CAVIAR combined the correlation statistic results and LD information to model and infer the probability that a variant was causal. This study assumed that only one causal variant existed per locus according to the parameter set -f1 -c1. We ranked the variants based on their causality probability given by CAVIAR, and the variant loci with a CAVIAR score greater than 0.1 were considered as fine-mapping loci.

### Enrichment analysis

We extracted CDSs, introns, 5’UTRs, 3’UTRs and intergenic regions from the gene annotation file (Ensembl version 97). All functional RNAs (eg. lncRNAs, scRNAs, mircoRNAs) were categorized as funcRNA regions. We also obtained the ATAC, TAD, and HIC compartment A/B annotation intervals from pig liver tissue data from the FR-AgENCODE project [31] (http://www.fragencode.org). In addition, we downloaded H3K4me3 and H3K27ac data from the liver tissues of three pigs under ENA accession number PRJEB6906 [32] and used MAC2 software [33] to identify the regions of promoters and enhancers in the pig genome. The significance of the enrichment of nominally significant eSTRs (SigSTRs, *P* ≤ 0.001), eSTRs (eSTRs, FDR < 1%) and fine-mapping eSTRs (FMeSTRs, FDR < 1% & CAVIAR score > 0.1) in the above mentioned genomic feature regions was determined by using Fisher’s exact test in the R program.

### Linkage disequilibrium between GWAS SNPs and STR

The Linkage disequilibrium (LD) between STRs and SNPs was estimated from the square of the correlation coefficient between the STR dosage and the SNP genotypes. Among them, the genotype of an SNP was coded as 0, 1, or 2, and the STR dosage was the sum of GB tags (GB = allele length – reference length) of the two alleles. We downloaded the complete QTL data of pigs from pigQTLdb [34] (https://www.animalgenome.org/cgi-bin/QTLdb/SS/index). QTL regions of less than 2 Mb were considered candidate fine-mapping QTL regions, and the merged QTL regions totalled 991 Mb after the redundant areas were deleted. Then, we compared the overlap between the pig eSTR and QTL regions. We also downloaded the information of a total of 15,736 pig GWAS SNPs from the ISwine database [35] (http://iswine.iomics.pro). We found that the mean LD of between STR and SNP drops below 0.1 when their distances surpass 100 kb. Therefore, we focused on eSTRs with a physical distance less than 100 kb from GWAS SNPs.

## Results

### STRs are significantly associated with liver gene expression traits in pigs

**Fig. 2:**
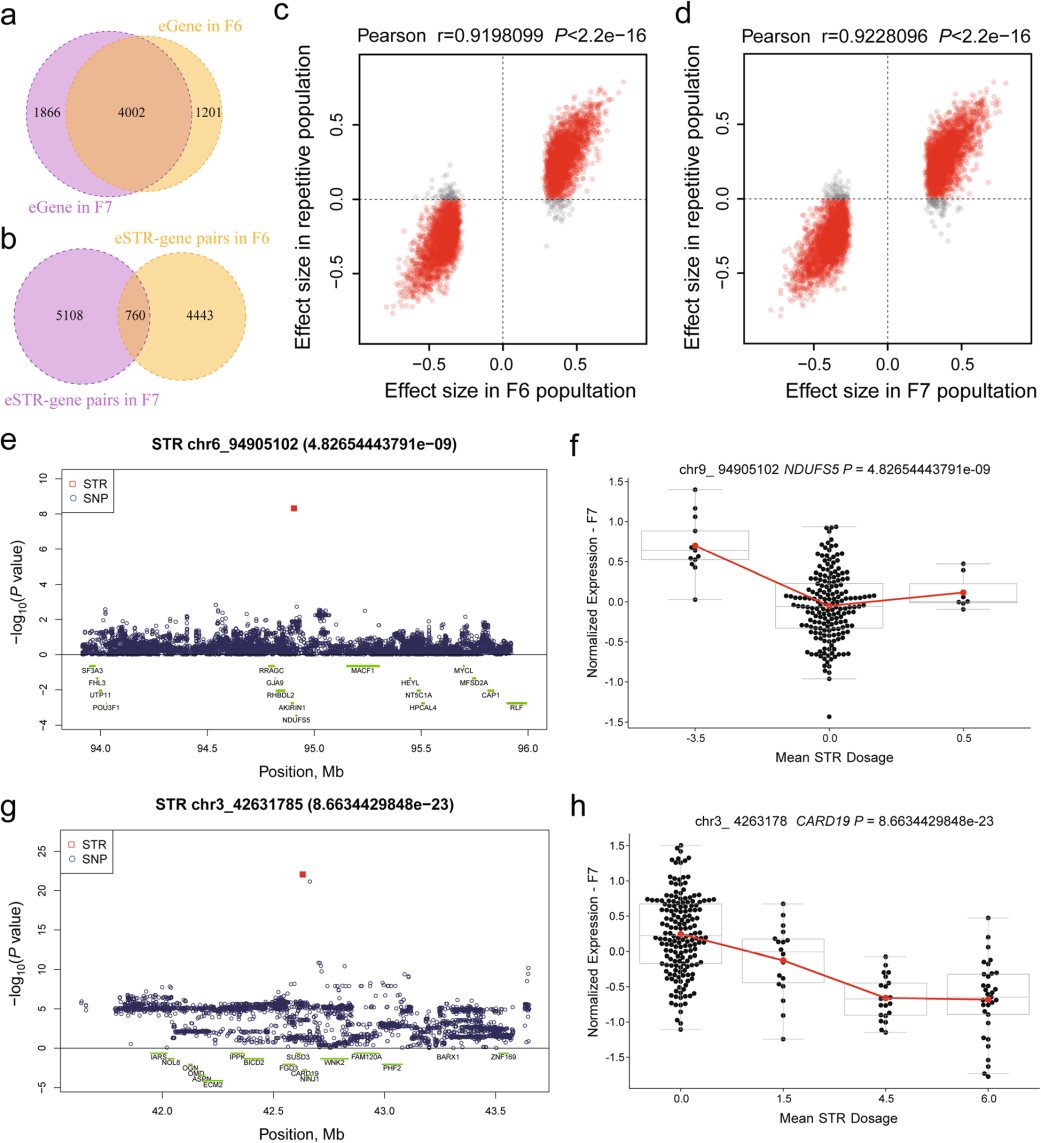
Results of genome-wide cis-eSTR analyses in the F6 and F7 population, respectively. (**a**) Unique and shared eSTRs between the F6 and F7 populations. (**b**) Unique and shared eGenes between the F6 and F7 populations. (**c**) and (**d**) Correlations between the effect sizes of eSTRs from F6 and F7, respectively. (**e**) and (**g**) Regional association plots for eSTRs that are more significant than nearby SNPs. (**f**) and (**h**) Relationship between STR dosage and gene expression

We first performed an association analysis between STR dosages and liver gene expression traits in the F6 and F7 populations, separately (Table 1). We focus on STRs within 1 Mb of the corresponding genes (Fig. 1). A total of 2,251,808 and 2,351,671 STR-gene pairs were tested in the F6 and F7 populations, respectively. We found 52,809 and 64,535 nominally significant eSTRs (SigSTR, *P* < 0.001) in F6 and F7, respectively. After multiple test corrections (eSTRs, FDR < 1%), we identified 5023 significant eSTRs in F6 (Additional file 3: Table S1), and 5868 significant eSTRs in the F7 population (Additional file 4: Table S2). The two populations shared 4002 eGenes and 760 eSTR-eGene pairs (Fig. 2a, b, Additional file 5: Fig. S3). The effect sizes of the eSTRs on the target genes in the F6 population showed a highly positive correlation (r = 0.92) with those in the F7 population, and vice versa (r = 0.923) (Fig. 2c, d). Similarly, the reciprocal π 1 statistics of the eSTRs in the two populations reached an average of 92% (Additional file 6: Fig. S4). The high replication rates supported the reliability of the identified STR-gene associations.

**Table 1:** An overview of eSTR analysis

	Total STRs	Nominal eSTRs	eSTR	FMeSTR
**F6 population**	137,348	52,809	5203	235
**F7 population**	143,098	64,535	5868	298
**Total**	149,481	79,186	8704	498

Notes: Nominal eSTRs (SigSTR, *P* ≤ 0.001), eSTR (eSTR, FDR < 1%) and fine-mapping eSTR (FMeSTR, FDR < 1% & CAVIAR score > 0.1)

We further tested whether the eSTRs remained significant after correcting for the SNP genotypes in the ±1 Mb regions around the corresponding genes. We observed that 1109 out of 5203 eSTRs (21.31%) in F6 pigs and 1375 out of 5868 (23.43%) eSTRs in F7 pigs remained significant after adjusting for SNP effects. On the other hand, we estimated the cis-heritability of each eGene and decomposed it as the sum of eSTR heritability and SNP heritability, and obtained an average eSTR heritability of 0.0885 (h_STR_) (Additional file 7: Fig. S5), corresponding to 27.5% of the total cis heritability of the gene expression traits.

To further compare the STRs with SNPs and InDels regarding their effects on gene expression traits, we used the same analysis pipeline to perform association analyses of SNPs and InDels with gene expression traits, separately in F6 and F7 pigs. Interestingly, we found that eSTRs were the lead variants for 164 genes in F6 individuals and 217 genes in F7 individuals, among which we identified twelve shared eGenes, and four eSTRs were found to be the lead variants (for *ABTB1*, *ASB5*, *NDUSF5* and *MPGI*) in both F6 and F7 individuals (Table 2). As examples, we present the eSTR signals of *NDUFS5* and *CARD19* in Fig. 2e–h and Additional file 8: Fig. S6a–6d.

**Table 2:** Lead eVariants are STR variation in both two population

Gene	F6 Population				F7 Population			
	Top STR	STR Motif	STR ***P*** value	Lead SNP ***P*** value	Top STR	STR Motif	STR ***P*** value	Lead SNP ***P*** value
***ABTB1***	chr13_71590920	AAAAAC	3.47E-27	2.67E-20	chr13_71590920	AAAAAC	2.63E-20	8.08E-19
***ASB5***	chr15_38935294	A	3.18E-16	2.74E-12	chr15_38935294	A	3.49E-17	1.64E-15
***NDUFS5***	chr6_94905102	T	9.74E-13	4.07E-07	chr6_94905102	T	4.83E-09	1.48E-03
***MPG***	chr3_40870942	GT	3.10E-06	5.14E-04	chr3_40870942	GT	4.54E-05	6.64E-05
***CARD19***	chr3_42640896	AT	3.16E-09	8.90E-08	chr3_42631785	AAC	8.66E-23	7.13E-22
***KNJ18***	chr12_61287890	AC	6.32E-09	2.82E-07	chr12_61381584	CTTTTT	3.10E-08	9.50E-08
***RTTN***	chr1_152545549	A	1.10E-07	7.15E-07	chr1_152565420	ATATG	1.50E-10	7.52E-10
***IL33***	chr1_215813132	T	4.50E-06	6.36E-05	chr1_215772135	A	2.23E-08	7.58E-07
***BCKDK***	chr3_17408094	GTTT	3.99E-12	1.56E-11	chr3_16931186	GGT	8.45E-08	8.64E-08
***RAB4B***	chr6_49976424	ACGGAG	3.10E-06	1.42E-05	chr6_48707226	A	1.10E-05	5.11E-05
***SNX33***	chr7_58078861	CGG	1.91E-08	1.46E-06	chr7_58006457	GT	2.13E-05	2.47E-05
***ARPC5***	chr9_124614177	A	2.76E-08	9.90E-08	chr9_124592786	GTT	6.38E-08	7.65E-08

**Fig. 3:**
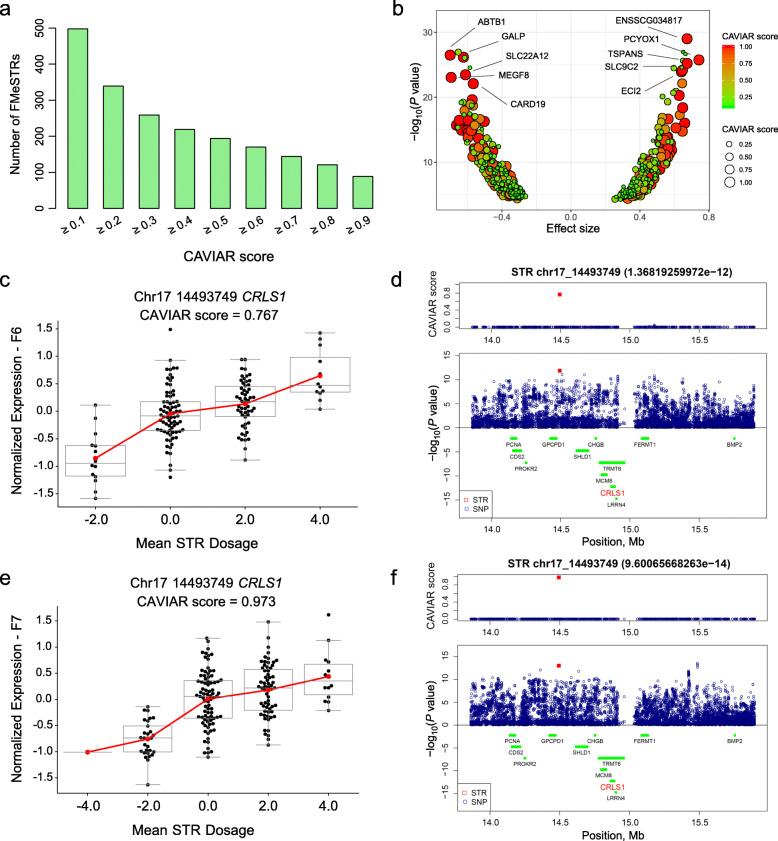
Results of 498 FMeSTRs in the F6/F7 populations. (**a**) Distribution of FMeSTRs in different CAVIAR score ranges. (**b**) Relationships among CAVIAR scores, log-transformed *P* values and effect sizes. (**c**) Boxplot of chr17:14493749-*CRLS1* gene pairs in the F6 population. (**d**) Dot plot of the *P* values and CAVIAR scores of chr17:14493749-*CRLS1* gene pairs in the F6 population. (**e**) Boxplot of chr17:14493749-*CRLS1* gene pairs in the F7 population. (**f**) Dot plot of the *P* values and CAVIAR scores of chr17:14493749-*CRLS1* gene pairs in the F7 population

We then used CAVIAR software to estimate the posterior probability that an eSTR is a potentially causal variant. A total of 498 eSTRs with probability scores > 0.1 were defined as fine-mapping eSTRs (FMeSTRs) including 235 FMeSTRs in F6 and 298 FMeSTRs in F7, respectively (Fig. 3a, Additional file 9: Table S3 and Additional file 10: Fig. S7). Among these FMeSTRs, we observed comparable numbers of positive and negative dosage effects of the FMeSTRs on the corresponding genes (Fig. 3b). FMeSTRs with higher CAVIAR scores tended to show more significant associations (−log_10_(*P* value)) and effect sizes for corresponding genes (Fig. 3b). We highlight the top 10 most significant FMeSTRs in Fig. 3b, including the FMeSTRs with negative dosage effects on *ABTB1, GALP, SLC22A12, MEGF8* and *CARD19*, and FMeSTRs with positive dosage effects on *ENSSCG034817, PCYOX1, TSPABS, SLC9C2* and *ECI2*. We found eighteen FMeSTR-gene pairs in both the F6 and F7 populations. We further illustrate the FMeSTR signal of *CRLS1* in Fig. 3c–f, which indicated that an (AAAC)n STR located on chr17:14493749 was the strongest eQTL for the expression of the *CRLS1* gene with a CAVIAR score greater than 0.767 in the F6/F7 populations. In summary, these results confirmed the significant influence of STRs on gene expression traits in pigs and identified a series of eSTRs and FMeSTRs for further analysis.

### Characterization of the distribution and enrichment of eSTRs in the genome

**Fig. 4:**
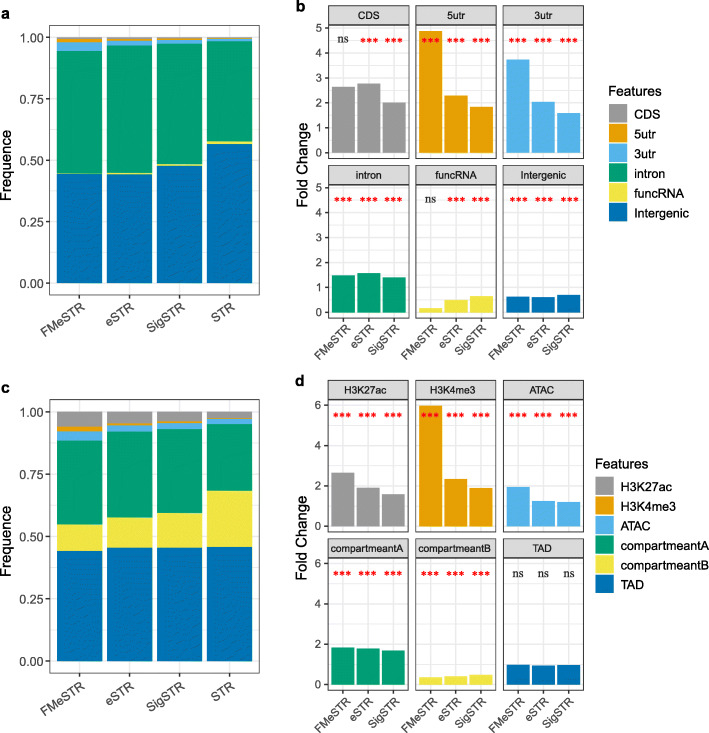
Enrichment analysis of the eSTR catalogue. (**a**) Distribution and (**b**) Enrichment analysis of eSTRs associated with different genomic features. SigSTR(*P* ≤ 0.001); eSTR (FDR ≤ 1%); FMeSTR (CAVIAR score > 0.1 & FDR ≤ 1%). (**c**) Distribution and (**d**) Enrichment analysis of eSTR associated with different epigenetic features. SigSTR(*P* ≤ 0.001); eSTR (FDR ≤ 1%); FMeSTR (CAVIAR score > 0.1 & FDR ≤ 1%)

We further investigated 498 FMeSTRs and 8704 eSTRs identified in the F6 and F7 populations for their overlap with different genome features (Fig. 4). Most of the eSTRs were distributed in intronic and intergenic regions (Fig. 4a). The enrichment analysis showed that the eSTRs and FMeSTRs were significantly enriched in 5UTR, CDS, 3UTR and intron regions, among which the strongest enrichment was observed in 5’UTRs (eSTR FC = 2.3, *P* = 8.6e− 08; FMeSTR FC = 4.8, *P* = 3.2e− 05), followed by CDS regions (eSTR FC = 2.8, *P* = 5.8e− 10; FMeSTR FC = 2.6, *P* = 2.0e− 01), 3’UTRs (eSTR FC = 2.0, *P* = 5.1e− 16; FMeSTR FC = 3.7, *P* = 1.8e− 08) and intron regions (eSTR FC = 1.6, *P* = 2.2e− 16; FMeSTR FC = 1.5, *P* = 1.3e− 05) (Fig. 4b). In contrast, FMeSTRs were significantly depleted in funcRNA regions (circRNA, lncRNA and snRNA). Moreover, we observed that eSTRs tended to be located in gene bodies, and their frequency decreased with their distance to their target genes (Additional file 11: Fig. S8). These results indicate that the closer the STR is to the target gene, the more likely it is to affect gene expression.

We further assembled a series of annotation data on the noncoding regions inferred from the epigenomic data of pig liver tissue (including H3K4me3, H3K27ac, ATAC, Topologically associated domain (TAD), HIC compartment A and HIC compartment B) obtained from public databases. We compared the distribution and enrichment of eSTRs/FMeSTRs in these functional areas, and the results showed that most FMeSTRs and eSTRs were located in active chromatin interaction areas (TAD and HIC compartment A) (Fig. 4c). Enrichment analysis showed that eSTRs and FMeSTRs were significantly enriched in H3K4me3, H3K27ac, HIC compartment A and ATAC regions (Fig. 4d), and underrepresented in HIC compartment B regions. It showed that approximately 36% of the eSTRs were within the same TAD region as the corresponding target gene (1821/5023 in F6 and 2089/5868 in F7, respectively). The density of eSTRs inside and outside TAD is unevenly distributed. The density inside TAD areas is about five times that outside these areas, indicating that STRs mainly function in areas where chromatin is open and active (Additional file 12: Fig. S9). Considering that eSTRs showed the strongest enrichment signals in H3K4me3 and H3K27ac modified markers, we speculate that the effects of eSTRs on gene expression traits may be exerted by altering the activity of promoters and enhancers.

### Identification of eSTRs as potential causal variants for complex traits

**Fig. 5:**
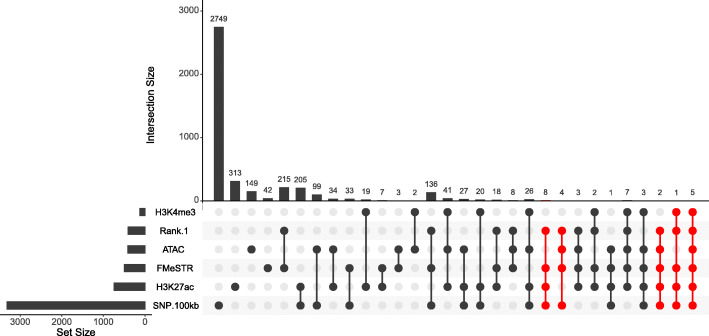
Number of eSTRs overlapping with FMeSTR, H3H4me3, ATAC and H3K27ac regions

**Fig. 6:**
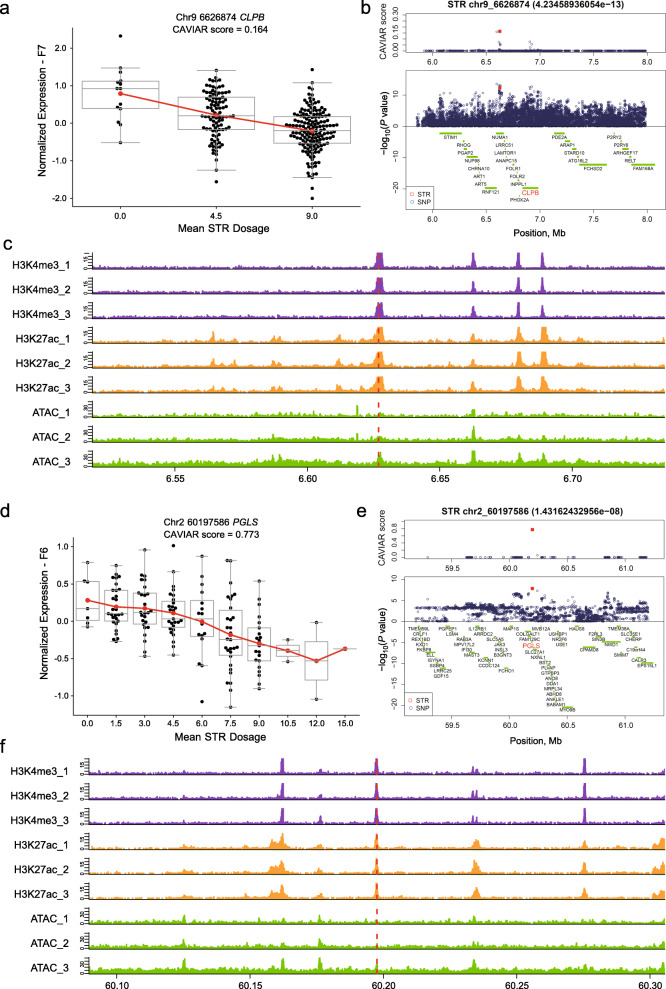
Colocalization of epigenetic peaks and eSTRs. Red dashed lines represent eSTR. Each kind of epigenetic marker was analysed in three parallel samples. (**a-c**) A (CCCCT) n STR related to *CLPB* gene expression is linked to GWAS SNPs and colocalizes with epigenetic peaks. (**d-f**) A (CCG) n STR related to *PGLS* gene expression is linked to GWAS SNPs and colocalizes with epigenetic peaks

Linkage disequilibrium among genetic markers is a key factor determining the power of genetic mapping studies. We used the square of the genotype correlation to estimate STR-SNP and SNP-SNP LD across the whole genome. The results show that the mean LD of STR-SNP was lower than that of SNP-SNP (Additional file 13: Fig. S10), suggesting that association studies based only on SNPs could overlook QTLs driven by STRs. We downloaded all SNP-trait data from pig GWASs available in public databases, including 8236 SNP loci for 499 phenotypic traits. We found that 3319 out of 8704 eSTRs were located in the upper and lower 100 kb regions of these GWAS SNPs. Among these eSTRs, 193 were FMeSTRs, with a top CAVIAR score greater than 0.1 (Fig. 5). Interestingly, we found that 20 FMeSTRs physically colocalized with at least one type of epigenetic peak (Fig. 5, Additional file 14: Table S4). For example, an FMeSTR on chromosome 9 fell in the conserved peaks of H3K4me3, H3K27ac and ATAC, which significantly affected the expression of the *CLPB* gene (Fig. 6a–c). The FMeSTR had a CAVIAR score of 0.164 in the f7 population, compared to 0.150 for the top SNP obtained using the same model. The nearby GWAS-SNPs were significantly related to hematocrit or the blood cell count [36], and the target eGene of the STR has also been reported to be associated with blood traits [37]. A similar case is that of an eSTR of the *PGLS* gene on chromosome 2, which is close to the GWAS loci for mean corpuscular hemoglobin content in Chinese Sutai pigs [36]. The FMeSTR had a CAVIAR score of 0.773 in the F6 population and was shown to colocalize with liver H3K4me3 and H3K27ac peaks (Fig. 6d–f). Notably, *PGLS* has been confirmed to be a key gene in the regulation of glycolysis and the pentose phosphate pathway, which are strongly related to cell metabolism [38], while its role in regulating MCHC remains to be validated. Other similar examples of eSTRs include: chr3_68578046(A), chr13_150219476(AT), chr6_71882310(GT), chr14_51443643(CGG), chr6_55078221(AAAAC) and chr7_4683910(AAAAT) (Additional file 14: Table S4, Additional file 15: Fig. S11a–11f). Taken together, these results suggest that one of the mechanisms of STRs may be to affect epigenetic modification activity, in turn affecting gene expression and complex traits.

## Discussion

Accumulating evidence has suggested that STRs are one of the most important contributors to the evolution of plants [39] and animals [40], and they also have an essential impact on gene expression and complex traits [9, 10, 41]. Here, we employed 556 liver tissue expression datasets and whole-genome sequencing datasets from two generations (F6 and F7) of a heterogeneous pig population to identify STRs that are significantly associated with the expression of nearby genes. In particular, we identified 235 and 298 fine-mapped eSTRs in the F6 and F7 populations, respectively. These eSTRs were enriched in regions such as the 5’UTRs, CDSs, and introns and in areas containing multiple epigenomic markers indicating the presence of promoters and enhancers. Moreover, we identified hundreds of published GWAS signals that were closely related to eSTRs according to both physical distance and functional annotations. Our results demonstrated that STRs contribute significantly to gene expression traits, and the generated eSTR catalogues will provide valuable resources for exploring the roles of STRs in complex traits in pigs.

Several studies have shown that STRs are enriched in 5’UTRs or promoter regions, which may be related to the functions of core promoters and transcription start sites during evolutionary selection in species [40, 42, 43]. This is consistent with our observation in the present study that eSTRs were most strongly enriched in 5’UTRs and H3K4me3 regions. In addition, we found that eSTR were enriched in ATAC and H3K27ac regions, suggesting that they may be a functional component of enhancers [8]. Only a small fraction of FMeSTRs were found to colocalize with epigenetic markers, suggesting that eSTR mediates the gene expression and complex traits through a variety of mechanisms. Among these FMeSTRs, the most abundant motifs were A/T, AC/GT, CCG/GGC, and other poly(A) N motifs (Additional file 16: Fig. S12). Moreover, CCG motifs were enriched in ATAC regions, H3K4me3 regions and 5UTR regions, while poly(A) motif were mainly enriched in H3K27ac regions, 3’UTRs, introns, Intergenic regions. Therefore, we assume that these STR could regulate gene expression through the following potential mechanisms. 1) Poly(A) sequences are a critical factor in nucleosome positioning. Long poly(A) repeats form an abnormal DNA structure, which abolishes nucleosome binding and alters gene expression levels [44]. 2) STRs regulate epigenetic properties such as DNA methylation patterns [45] and lead to the formation of heterochromatin [46]. 3) Moreover, we observed that 31 out of 498 fine-mapping eGenes encoded transcription factors according to the annotation information from AnimalTFDB (v3.0, [47]). Other studies have also confirmed that STRs can form transcription factor binding sites or regulate transcription factor binding efficiency [48–50]. 4) In addition, STRs affect regulatory element spacing, such as the distance between the enhancers and promoters or the distance between the promoters and transcription factor binding sites [51, 52]. 5) STRs cause the formation of unusual DNA secondary structures, including Z-DNA, H-DNA and G-quadruplexes. These structures may play a role in regulating supercoiling during transcriptional regulation [53–55]. For example, the hairpin structures and the G-quadruplex structures have been shown to regulate alternative splicing. Possibly by changing the distance between elements during splicing or hindering the progress of RNA polymerase [56]. In summary, STR variation may regulate gene expression and control complex traits through different genetic mechanisms and biological pathways.

Although we have provided the first dataset of eSTRs in pigs, which were repeatedly verified in both the discovery F6 population and validation F7 population, the present work inevitably has some shortcomings. We found that STRs affect the intermediate phenotype, namely gene expression, but there is no direct evidence to explain which complex traits or diseases are significantly associated with the STRs in our dataset. Moreover, the mechanism whereby STRs affect gene expression in pigs is still unclear. These are the directions that we will focus on and strive to investigate in the future.

## Conclusions

In this study, we first identified 5023 and 5868 significant eSTRs, as well as 235 and 298 fine-mapping eSTRs, in pig liver tissues from our F6 and F7 populations, respectively. Interestingly, some of these eSTRs were independent of eSNP effects, suggesting that these STR variations may also be causal mutations. Second, we found that fine-mapped eSTR were significantly enriched in CDSs, UTRs, intron regions, and epigenetic modification regions such as ATAC, HIC Compartment A and H3K4me3 regions, in the pig genome, which may be related to the different regulatory mechanisms of eSTRs. Finally, we observed associations between several hundred eSTRs and GWAS SNPs. Among these associations, 20 STRs were physically colocalized with epigenetic peaks, suggesting that these STRs are more likely to be candidate causal variants. In conclusion, this study provides a comprehensive and systematic analysis of eSTRs in porcine liver tissues of heterogeneous populations, which is valuable for dissecting the roles of STRs underlying the variations in complex traits in pigs.

## Supplementary Information

**Additional file 1 Fig. S1.** The workflow of eSTR analysis in pigs. WGS, Whole-genome sequencing

**Additional file 2 Fig. S2**. The call rate of genome-wide STR genotypes among 556 pigs from heterogeneous population

**Additional file 3 Table S1.** The 5203 eSTRs in the F6 population

**Additional file 4 Table S2.** The 5868 eSTRs in the F7 population

**Additional file 5 Fig. S3.** eSTRs showing significant associations with gene expression

**Additional file 6 Fig. S4.** Replication ratio based on π1 statistics estimated with the qvalue package. (a) Estimation of π1 statistics with the F6 term as the discover population and F7 as the replication population. (b) Estimation of π1 statistics with the F7 term as the discover population and F6 as the replication population

**Additional file 7 Fig. S5.** Heritability estimates for eSTRs in cis-regions

**Additional file 8 Fig. S6.** Two examples of eSTR-gene association analyses in the F6 population. (a) and (c) Regional association plots for eSTRs that are more significant than nearby SNPs. (b) and (d) Relationship between STR dosage and gene expression

**Additional file 9 Table S3.** The 498 FMeSTRs in the F6/F7 population

**Additional file 10 Fig. S7.** FMeSTRs showing significant associations with gene expression

**Additional file 11 Fig. S8.** Distribution of eSTRs distances to the closest gene body

**Additional file 12 Fig. S9.** Distribution of eSTRs located in TAD or non-TAD regions

**Additional file 13 Fig. S10.** Linkage disequilibrium decay analysis. The LD between SNPs and STRs was evaluated based on the square of the Pearson correlation coefficient

**Additional file 14 Table S4.** STRs linked with GWAS SNPs are located in epigenetic peaks

**Additional file 15 Fig. S11.** Co-localization of epigenetic peaks and eSTR. Red dashed lines represent eSTR. Each kind of epigenetic marker was analysed in three parallel samples. (a-c) A (CCG) n STR related to the expression of the *PSMD6* gene is linked to GWAS SNPs and colocalizes with epigenetic peaks. (d-f) A (AAAC) n STR related to *DHDH* gene expression is linked to GWAS SNPs and colocalizes with epigenetic peaks

**Additional file 16 Fig. S12.** Major motif components in FMeSTRs

## Data Availability

Scripts and supplemental datasets used in this study are available from: https://github.com/jxlabWzZ/Sus_Liver_eSTRs. The raw sequencing data are available upon request.

## Abbreviations

ATAC: Assay for transposase accessible chromatin with high-throughput sequencing
eQTL: Expression quantitative trait locus
eSNP: Expression SNP.
eSTR: Expression STR.
FDR: False discovery rate.
FMeSTR: Fine-mapping expression STR.
FPKM: Fragment per kiloBase per million mapped reads.
HIC: High-through chromosome conformation capture.
LD: Linkage disequilibrium.
SigSTR: Nominally significant eSTRs.
SNP: Single nucleotide polymorphism.
STR: Short tandem repeat.
TAD: Topologically associated domain.
