# Supplementary figures and images for: Mapping short tandem repeats for liver gene expression traits helps prioritize potential causal variants for complex traits in pigs
Source: J Anim Sci Biotechnol. 2022 Jan 17;13:8. doi: 10.1186/s40104-021-00658-z (PMC8762894; doi:10.1186/s40104-021-00658-z)

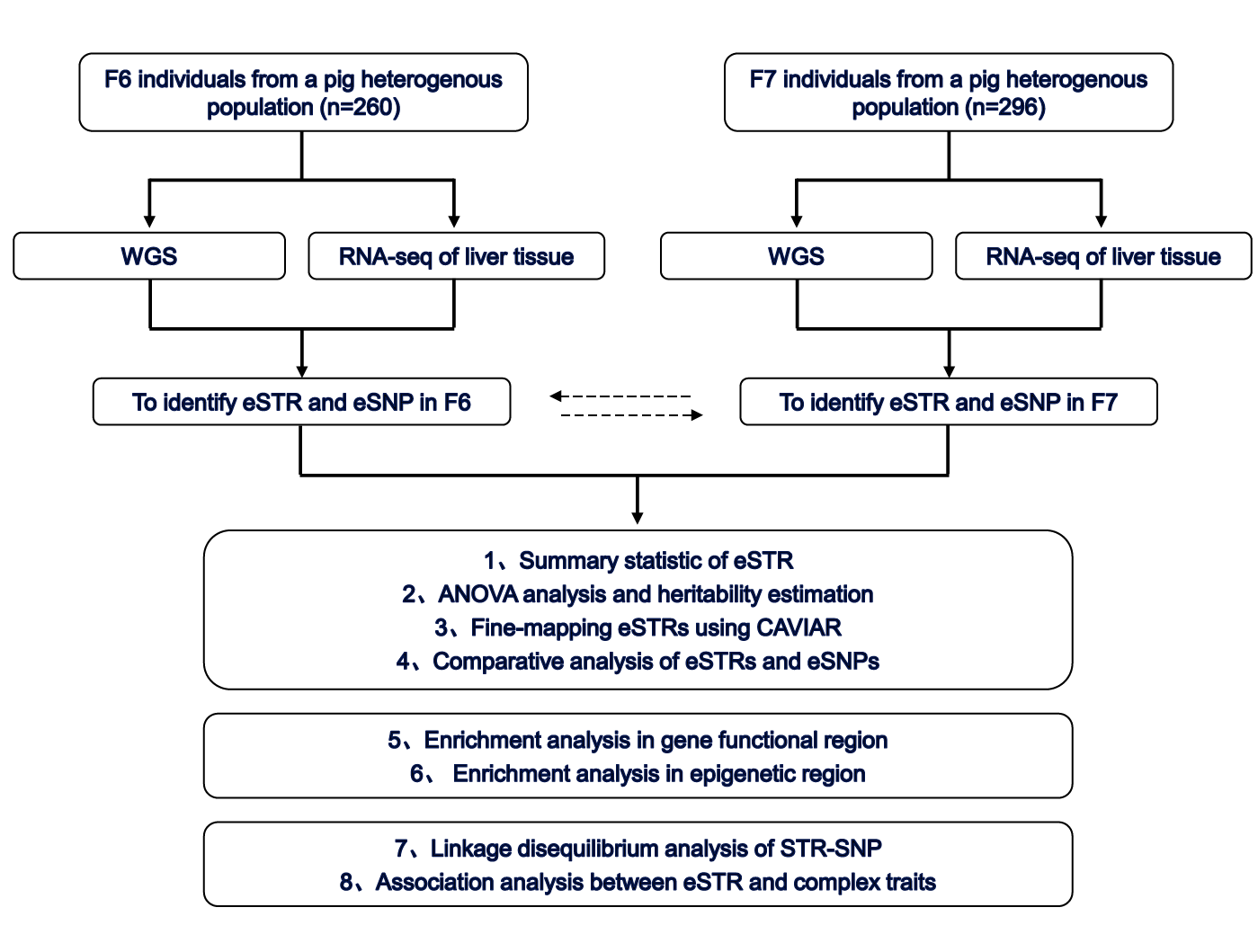

Supplement: Supplementary file 1 — Additional file 1 Fig. S1. The workflow of eSTR analysis in pigs. WGS, Whole-genome sequencing [file 40104_2021_658_MOESM1_ESM.png]

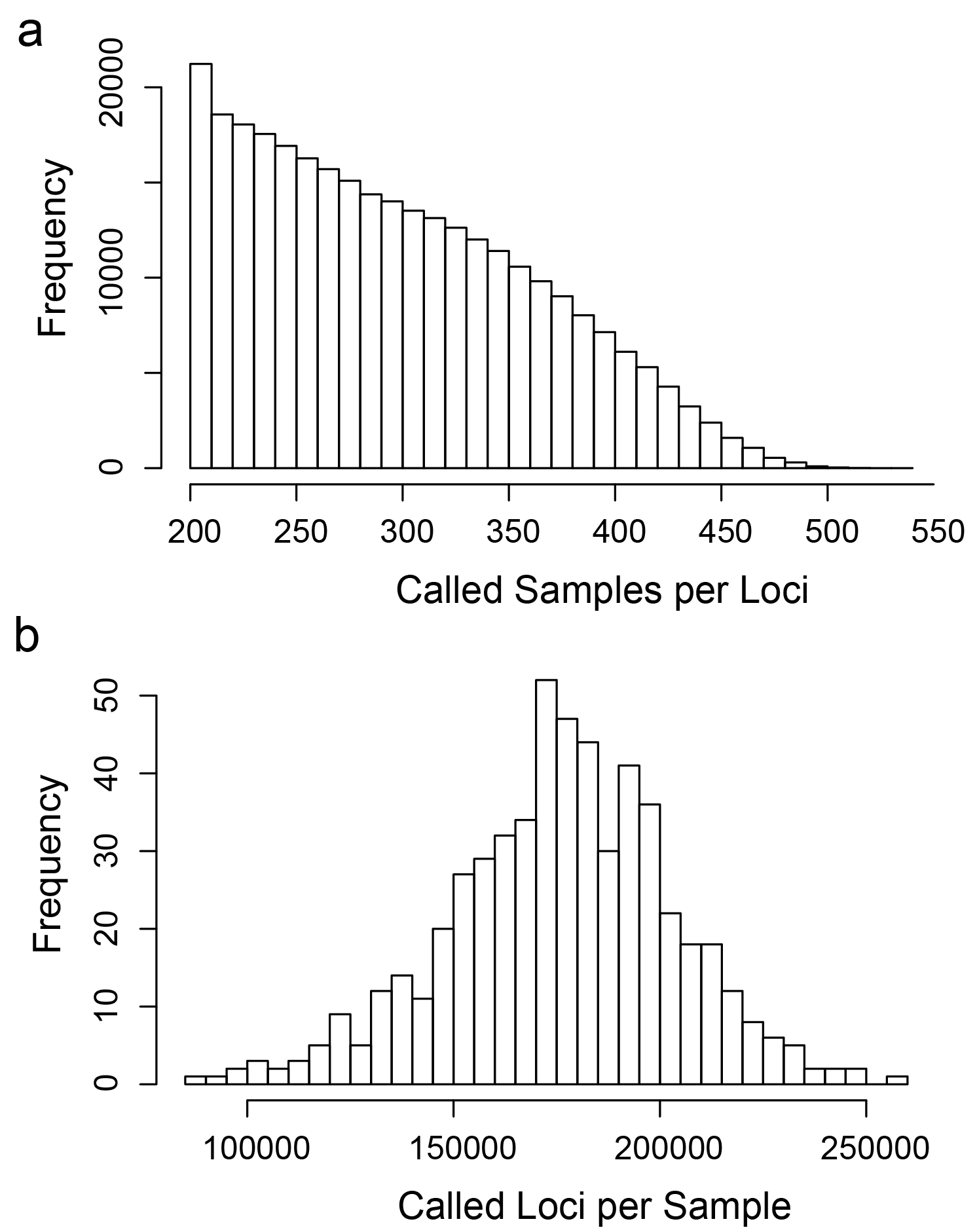

Supplement: Supplementary file 2 — Additional file 2 Fig. S2. The call rate of genome-wide STR genotypes among 556 pigs from heterogeneous population [file 40104_2021_658_MOESM2_ESM.png]

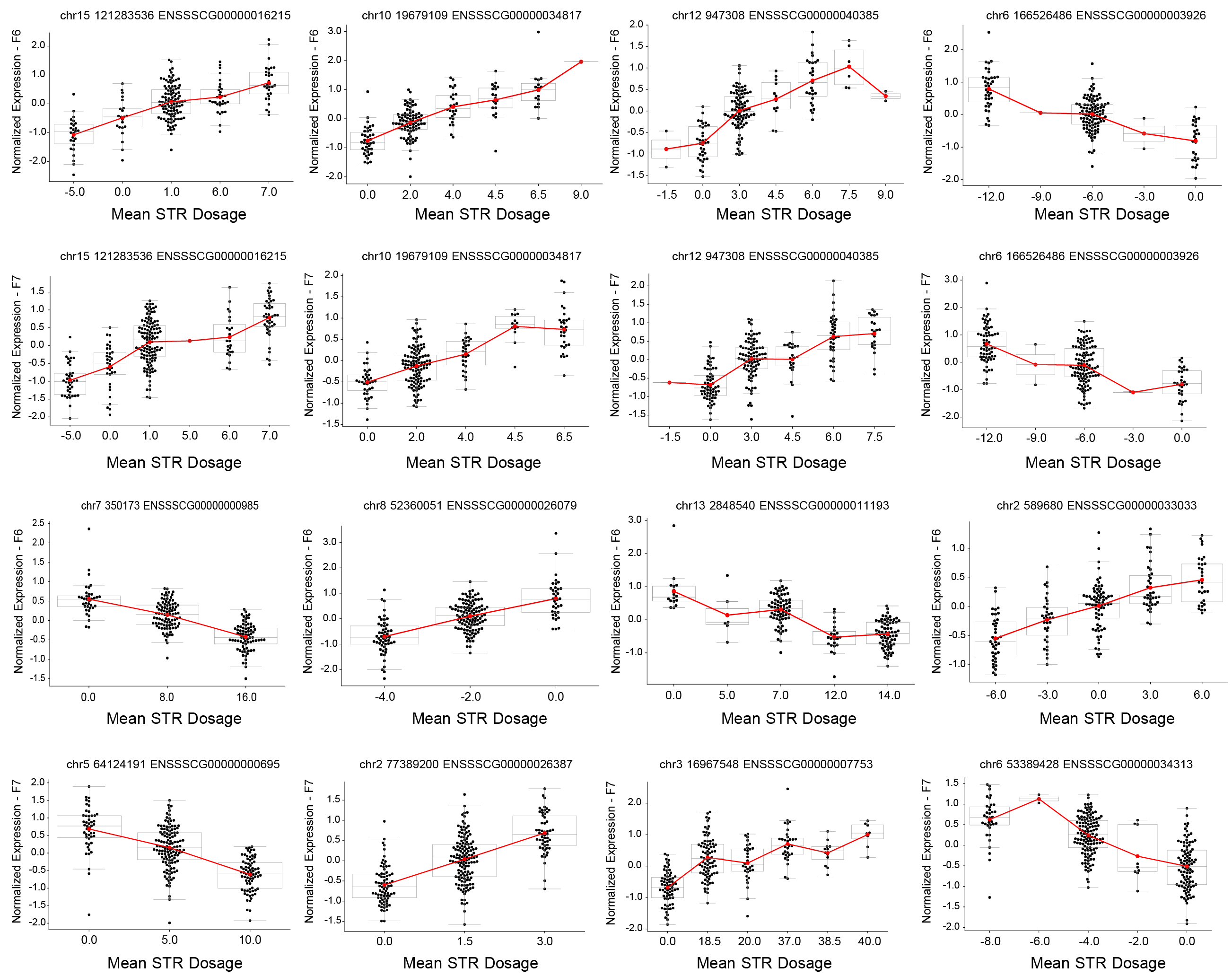

Supplement: Supplementary file 5 — Additional file 5 Fig. S3. eSTRs showing significant associations with gene expression [file 40104_2021_658_MOESM5_ESM.png]

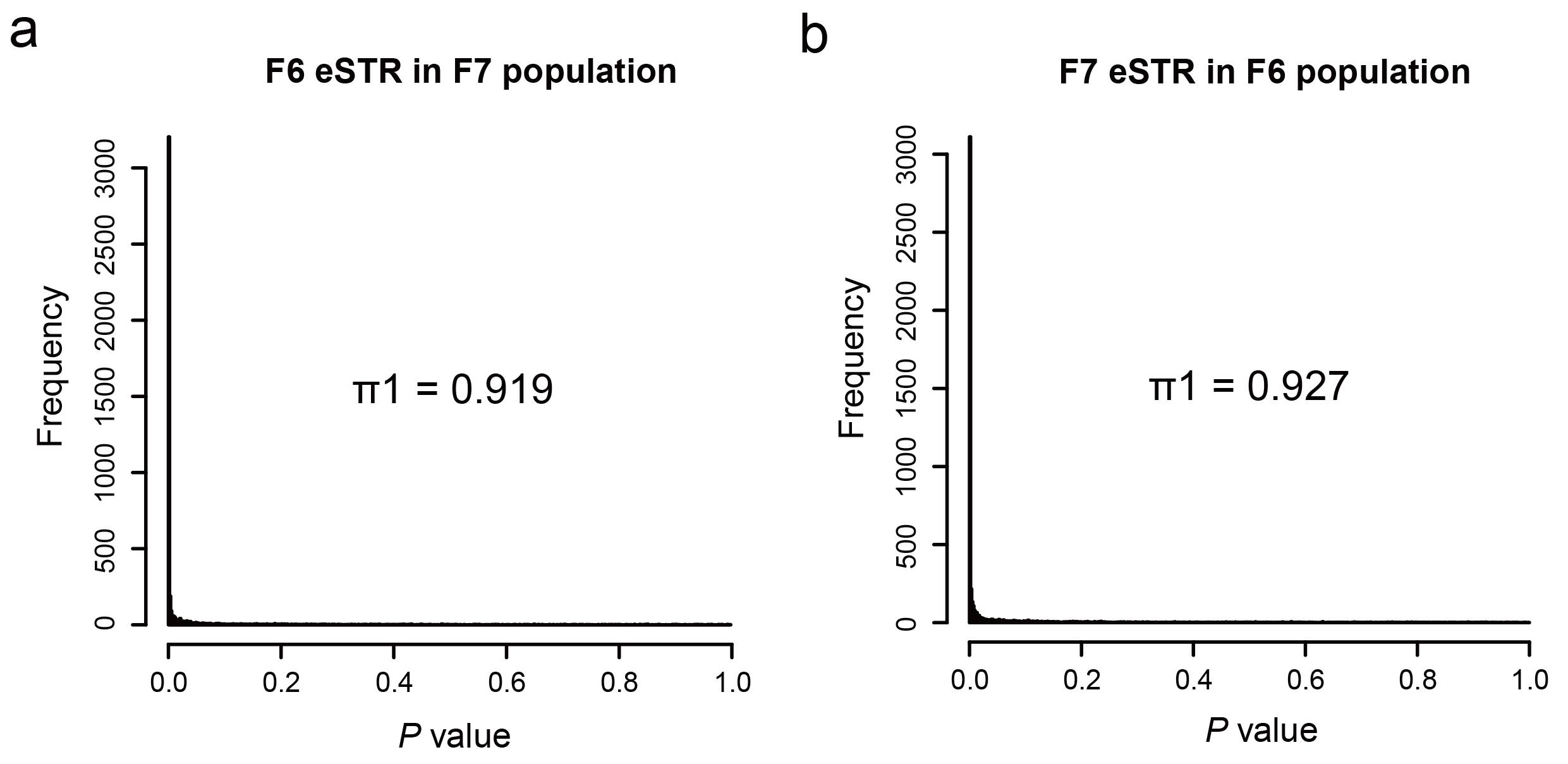

Supplement: Supplementary file 6 — Additional file 6 Fig. S4. Replication ratio based on π1 statistics estimated with the qvalue package. (a) Estimation of π1 statistics with the F6 term as the discover population and F7 as the replication population. (b) Estimation of π1 statistics with the F7 term as the discover population and F6 as the replication population [file 40104_2021_658_MOESM6_ESM.png]

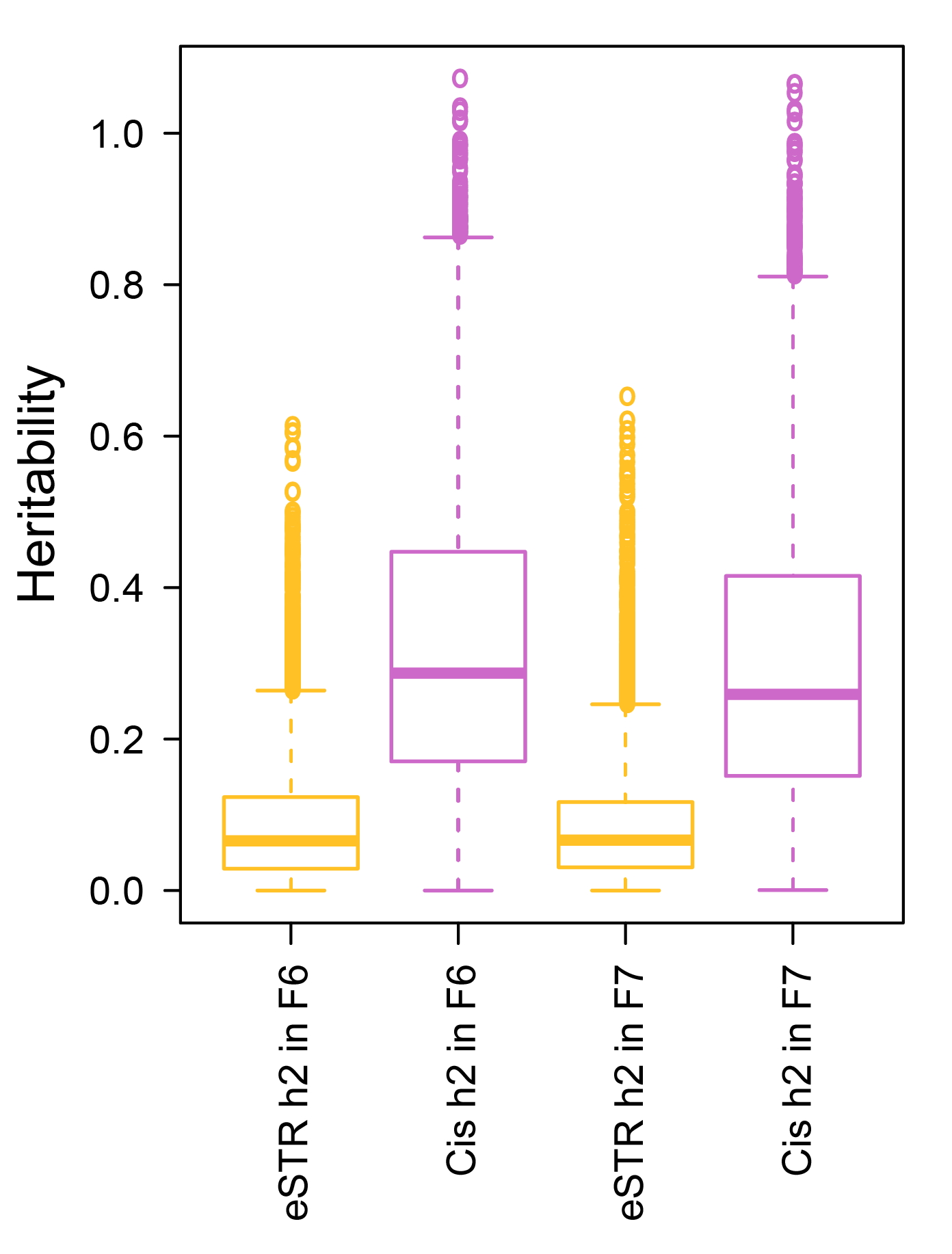

Supplement: Supplementary file 7 — Additional file 7 Fig. S5. Heritability estimates for eSTRs in cis-regions [file 40104_2021_658_MOESM7_ESM.png]

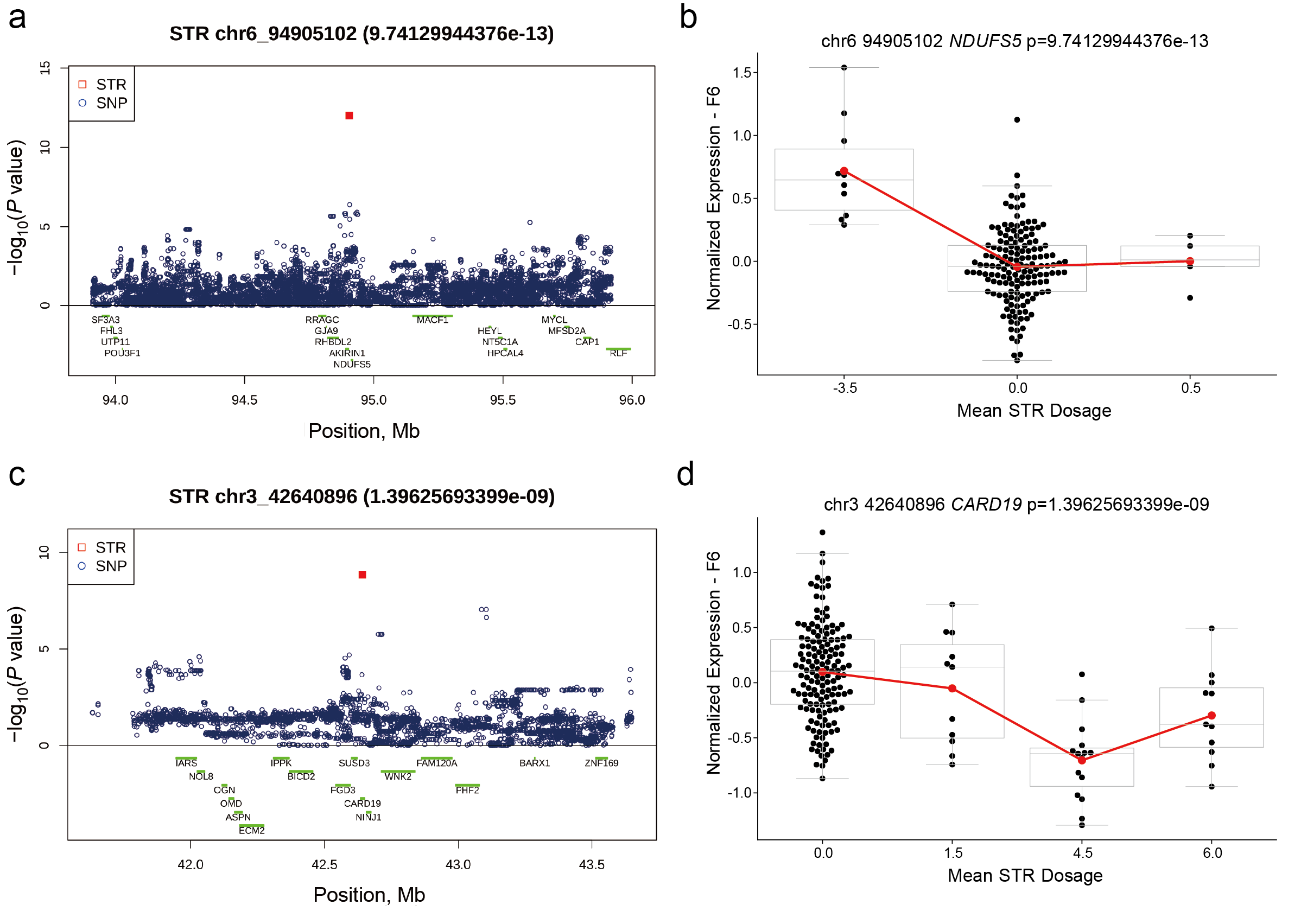

Supplement: Supplementary file 8 — Additional file 8 Fig. S6. Two examples of eSTR-gene association analyses in the F6 population. (a) and (c) Regional association plots for eSTRs that are more significant than nearby SNPs. (b) and (d) Relationship between STR dosage and gene expression [file 40104_2021_658_MOESM8_ESM.png]

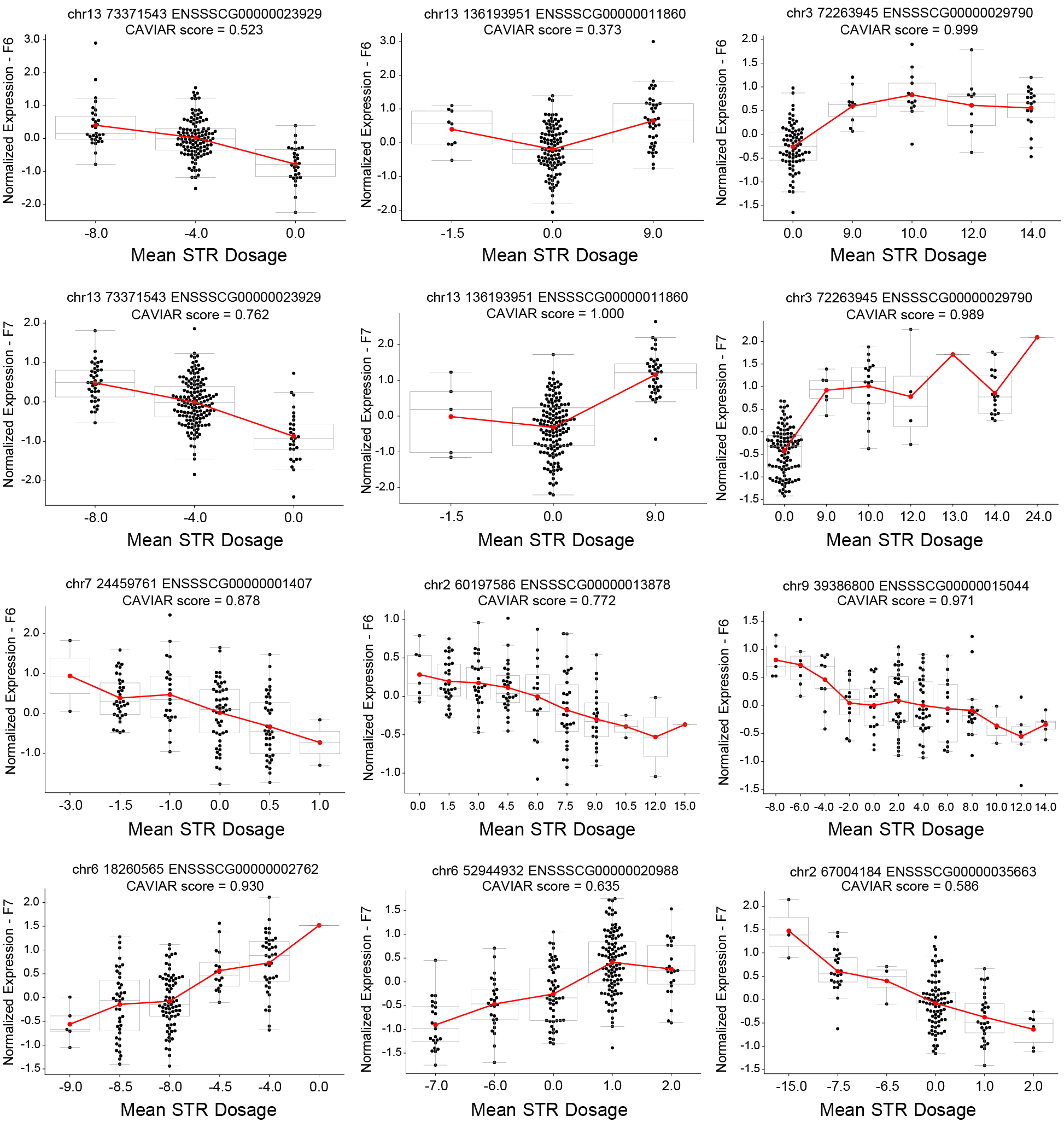

Supplement: Supplementary file 10 — Additional file 10 Fig. S7. FMeSTRs showing significant associations with gene expression [file 40104_2021_658_MOESM10_ESM.png]

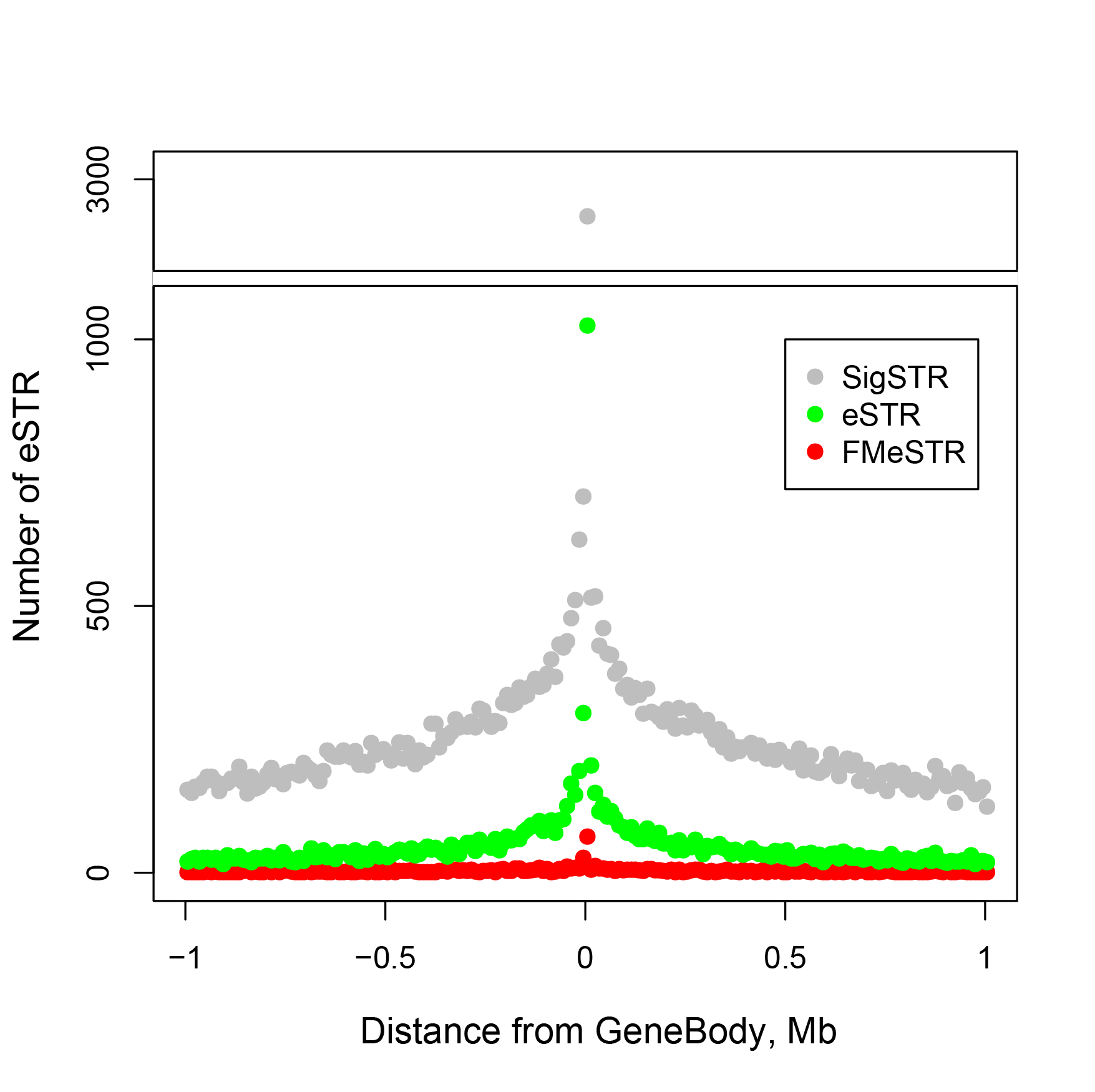

Supplement: Supplementary file 11 — Additional file 11 Fig. S8. Distribution of eSTRs distances to the closest gene body [file 40104_2021_658_MOESM11_ESM.png]

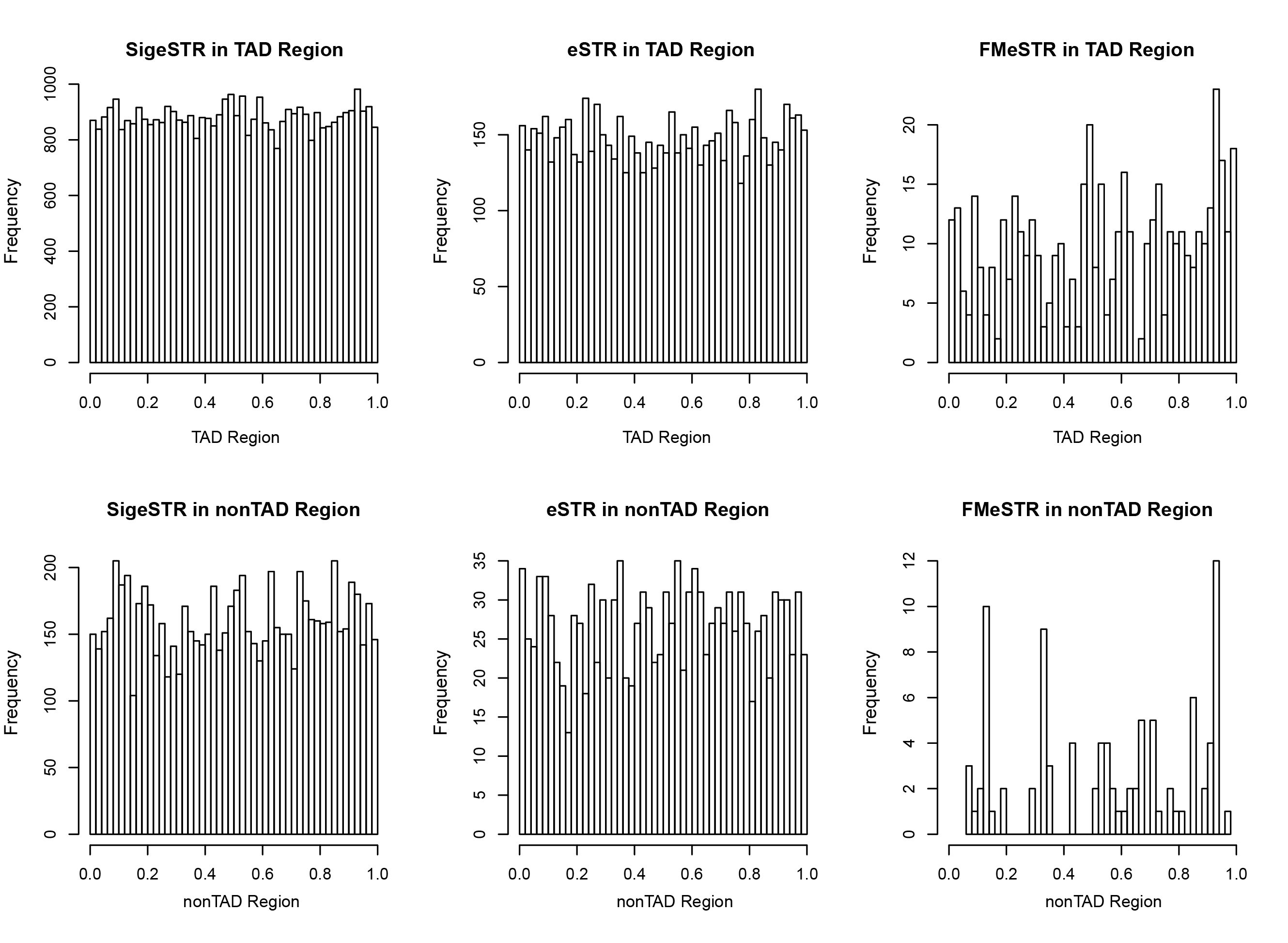

Supplement: Supplementary file 12 — Additional file 12 Fig. S9. Distribution of eSTRs located in TAD or non-TAD regions [file 40104_2021_658_MOESM12_ESM.png]

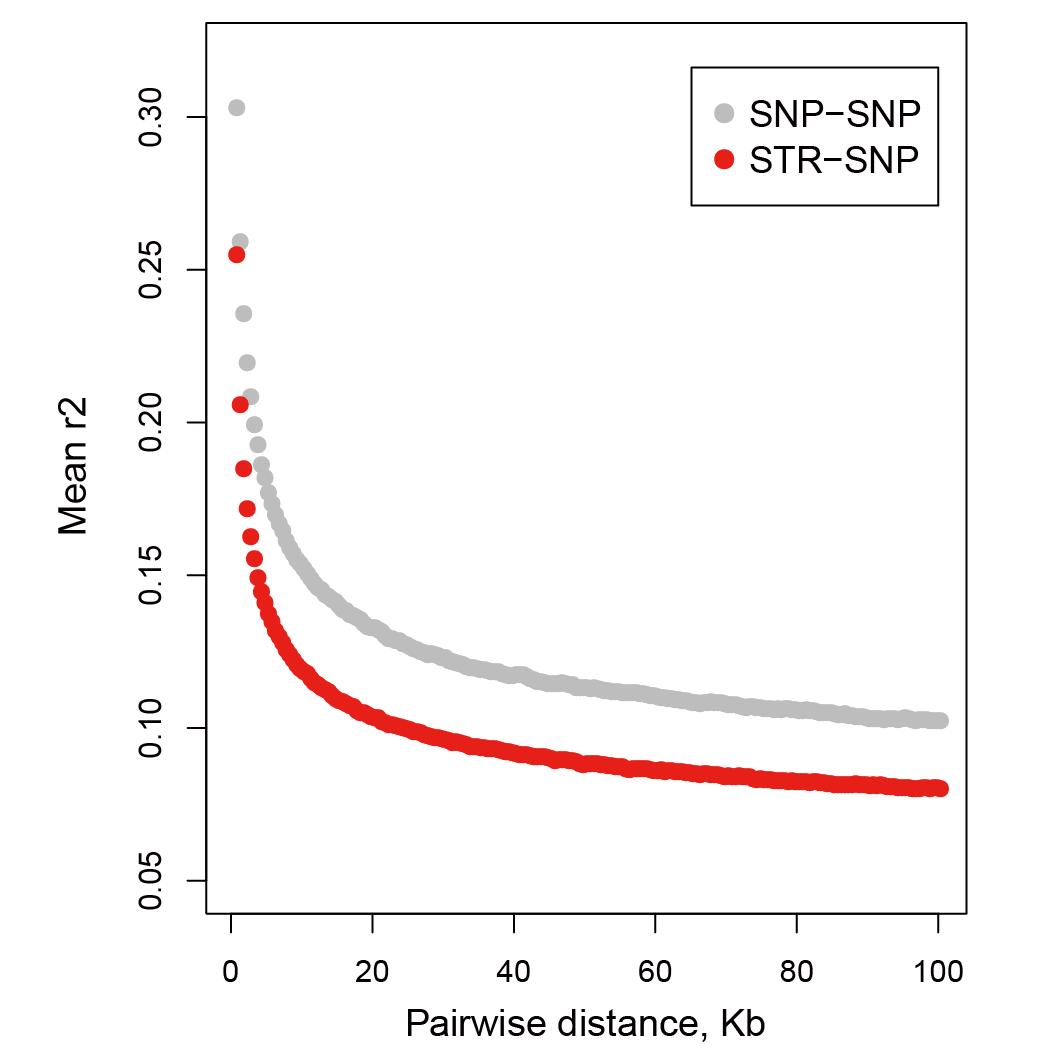

Supplement: Supplementary file 13 — Additional file 13 Fig. S10. Linkage disequilibrium decay analysis. The LD between SNPs and STRs was evaluated based on the square of the Pearson correlation coefficient [file 40104_2021_658_MOESM13_ESM.png]

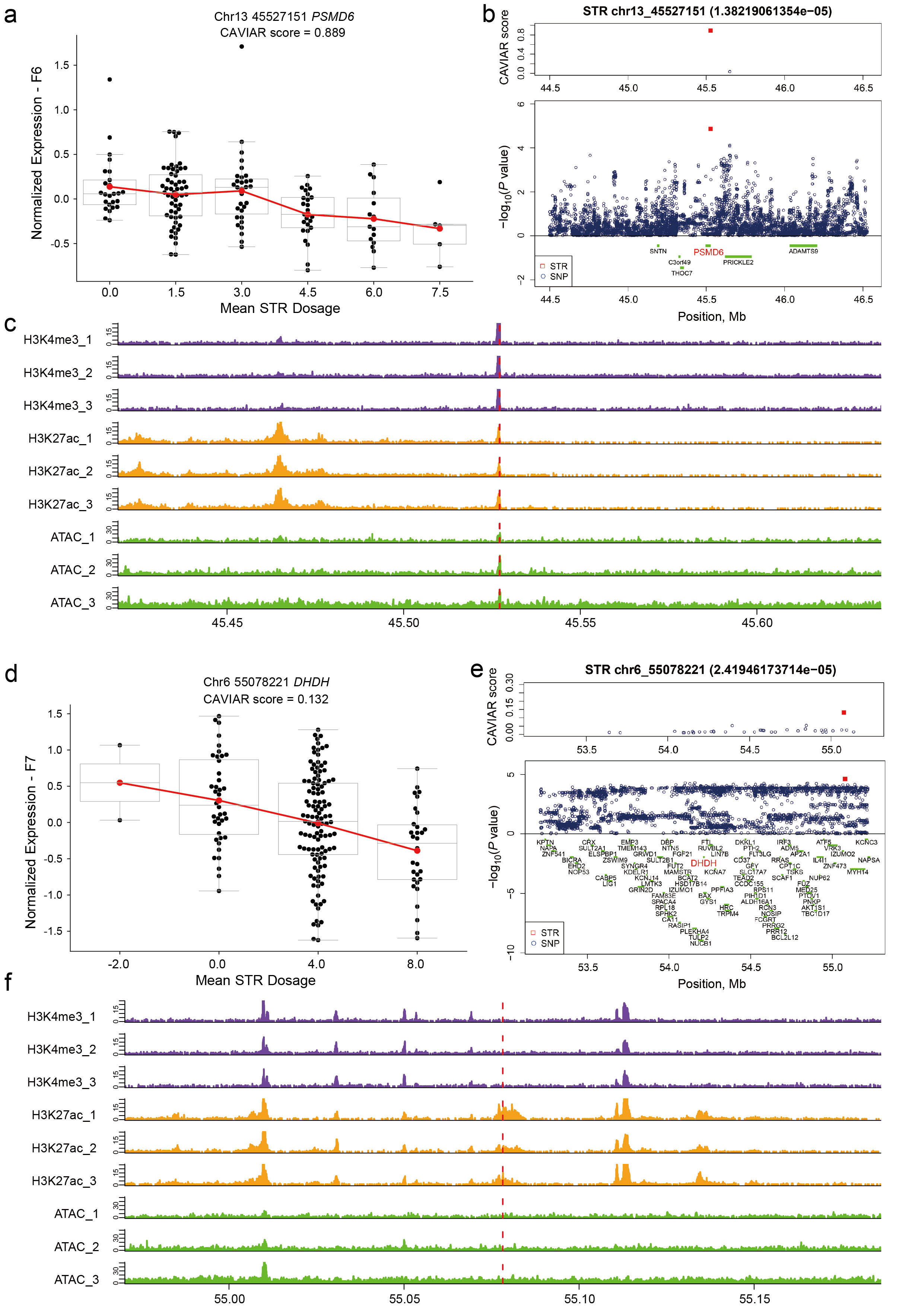

Supplement: Supplementary file 15 — Additional file 15 Fig. S11. Co-localization of epigenetic peaks and eSTR. Red dashed lines represent eSTR. Each kind of epigenetic marker was analysed in three parallel samples. (a-c) A (CCG) n STR related to the expression of the PSMD6 gene is linked to GWAS SNPs and colocalizes with epigenetic peaks. (d-f) A (AAAC) n STR related to DHDH gene expression is linked to GWAS SNPs and colocalizes with epigenetic peaks [file 40104_2021_658_MOESM15_ESM.png]

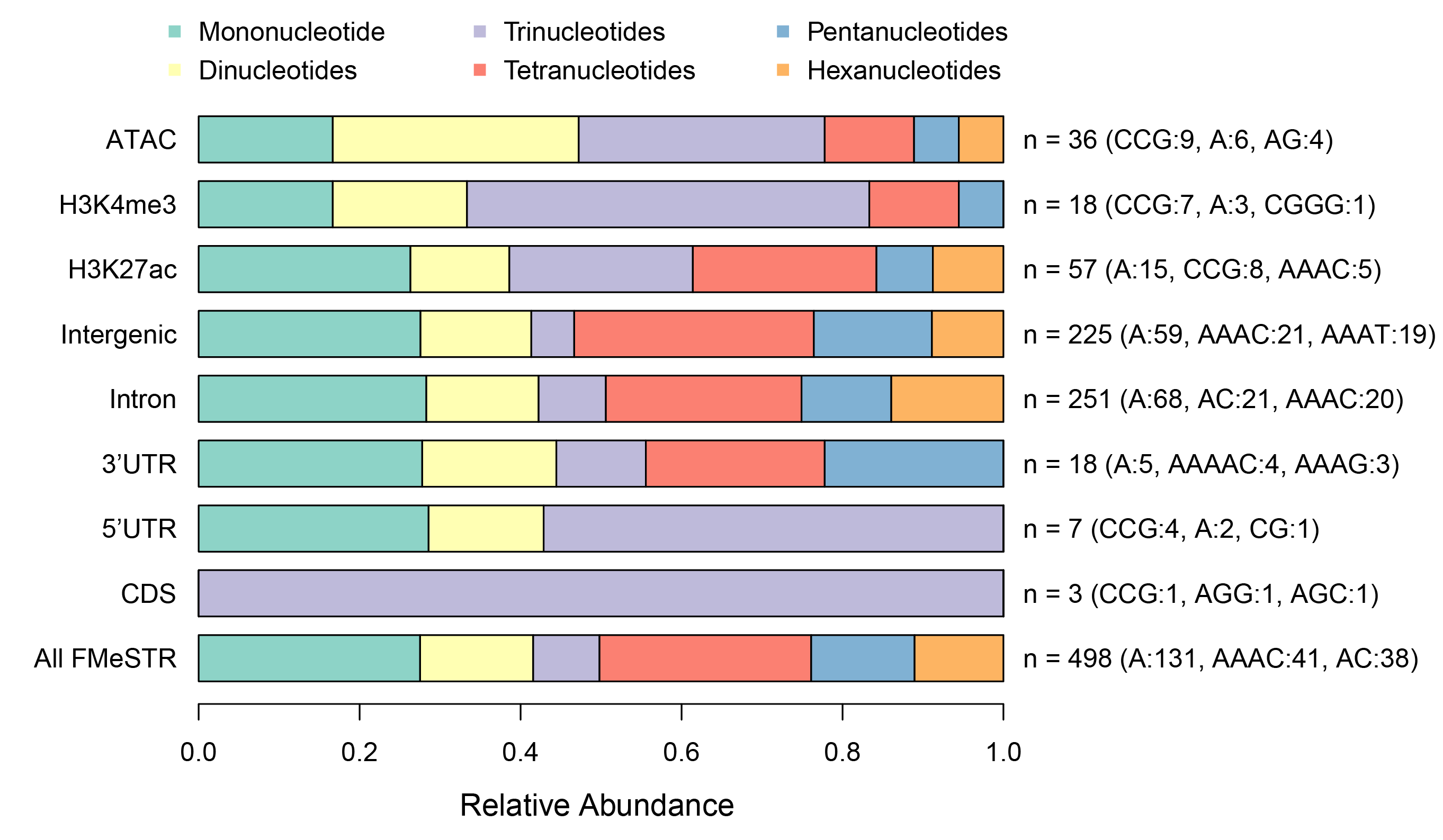

Supplement: Supplementary file 16 — Additional file 16 Fig. S12. Major motif components in FMeSTRs [file 40104_2021_658_MOESM16_ESM.png]
